# Harnessing Microfluidics for the Effective and Precise Synthesis of Advanced Materials

**DOI:** 10.3390/mi16101106

**Published:** 2025-09-28

**Authors:** Xinlei Qi, Guoqing Hu

**Affiliations:** Department of Engineering Mechanics, State Key Laboratory of Fluid Power and Mechatronic Systems, Zhejiang University, Hangzhou 310027, China; xqi@zju.edu.cn

**Keywords:** microfluidics, advanced materials, precise synthesis, particle

## Abstract

Microfluidic methods are powerful platforms for synthesizing advanced functional materials because they allow for precise control of microscale reaction environments. Microfluidics manipulates reactants in lab-on-a-chip systems to enable the fabrication of highly uniform materials with tunable properties, which are crucial for drug delivery, diagnostics, catalysis, and nanomaterial design. This review emphasizes recent progress in microfluidic technologies for synthesizing functional materials, with a focus on polymeric, hydrogel, lipid-based, and inorganic particles. Microfluidics provides exceptional control over the size, morphology, composition, and surface chemistry of materials, thereby enhancing their performance through uniformity, tunability, hierarchical structuring, and on-chip functionalization. Our review provides novel insights by linking material design strategies with fabrication methods tailored to biomedical applications. We also discuss emerging trends, such as AI-driven optimization, automation, and sustainable microfluidic practices, offering a practical and forward-looking perspective. As the field advances toward robust, standardized, and user-friendly platforms, microfluidics has the potential to increase industrial adoption and enable on-demand solutions in nanotechnology and personalized medicine.

## 1. Introduction

Advanced materials at the micro- and nanoscale hold immense potential across a wide range of scientific and engineering fields, including drug delivery and screening [1,2,3], disease prevention, diagnostics and treatment in biomedicine [4], and the synthesis of novel organic and inorganic materials [5,6,7,8]. Key material properties—such as size, size distribution, shape and surface chemistry—are critical determinants of material behavior, a role that is particularly pronounced in precision micro- and nanoscale systems [9,10,11]. In nanoparticle-based drug delivery, for instance, parameters such as particle size, shape and surface charge dictate biodistribution among organs like the lungs, liver, spleen, and kidneys [12]. Particle size also influences the in vivo clearance rate, thereby affecting the residence time of nanoparticles in the bloodstream. A prolonged and optimal circulation duration is necessary to ensure that nanoparticles have sufficient opportunity to accumulate at the intended target site. During interactions with cellular membranes, nanoparticles are internalized via pathways including phagocytosis, endocytosis, and pinocytosis, followed by intracellular trafficking [13]. Particle shape is a critical physicochemical factor that modulates these uptake mechanisms and significantly influences the efficiency and specificity of drug delivery [2]. Optimization of these physicochemical properties is crucial for designing effective nanomedicines that exploit the enhanced permeation and retention (EPR) effect [2], thereby enabling more accurate and efficacious tumor-targeted therapies [14]. Therefore, precise and efficient synthetic methods are indispensable for controlling these material characteristics. However, conventional methods are often limited in their ability to produce such advanced materials. Macroscale mixing, for example, frequently lacks the resolution to control local reactant concentrations precisely, which adversely affects material properties and leads to undesirable polydispersity.

The advancement of microfluidic technology has introduced a powerful and versatile platform for the controlled synthesis of precision materials [15,16,17,18,19,20]. By utilizing micro- to nanoscale channels as confined reaction environments, these systems enable continuous-flow synthesis through techniques such as controlled pumping. Additionally, the optical transparency of microfluidic chip materials allows for real-time visualization and precise manipulation of the reaction process [21,22]. These features make it particularly well-suited for the synthesis of advanced micro- and nanomaterials from complex or costly precursors [23]. Unlike traditional bulk stirring, microfluidics employs laminar flow to mix reactants, which establishes stable and predictable flow and concentration profiles [24]. In such systems, reactant mixing is governed by diffusion-dominated transport, leading to more stable, efficient, and controllable reactions. Furthermore, microfluidic platforms exhibit a high surface-area-to-volume ratio that facilitates rapid heat and mass transfer, enhancing reaction kinetics and uniformity [25]. These methods offer unprecedented, microscale control over reagent concentration, reaction time, and temperature, allowing for uniform reactant distribution and greater tunability than conventional approaches [26]. As a form of lab-on-a-chip technology, microfluidics enables high-throughput screening of diverse reaction conditions using minimal reagent volumes [27], making it ideal for the synthesis of novel materials where reduced reagent consumption and compatibility with precious or sensitive precursors are critical [28]. These attributes position microfluidics as a transformative tool for next-generation material synthesis [29].

Microfluidic technology is a powerful platform for synthesizing advanced functional materials, offering distinct advantages in fabricating polymers [30], hydrogel microparticles [31,32], and nanoparticles [33,34]. Droplet-based microfluidics provides a highly controllable reaction environment where droplet size can be precisely tuned by adjusting the flow rates of immiscible fluids, enabling the generation of monodisperse particles with excellent uniformity [35,36,37]. This relationship between droplet size and flow parameters can be modeled quantitatively to support process optimization. By engineering microfluidic systems, it is possible to exert fine control over particle morphology and composition. In nanoparticle synthesis, continuous-flow microfluidic reactors facilitate rapid and homogeneous mixing, which significantly improves particle size uniformity, reproducibility, and batch-to-batch consistency [38,39]. For instance, lipid nanoparticles (LNPs)—crucial carriers in drug and gene delivery that are highly sensitive to production conditions—benefit from the precise and rapid mixing afforded by microfluidics [40], which surpasses conventional bulk methods and ensures LNP stability, dispersity, and therapeutic efficacy [41]. Microfluidics also advances the fabrication of multifunctional and hierarchical materials. Surface functionalization, such as the introduction of targeting ligands, polyethylene glycol (PEG) chains [42], or fluorescent tags, can be achieved in situ during synthesis or via on-chip post-processing to endow particles with diverse functionalities.

This review focuses on the efficient and precise synthesis of advanced materials using microfluidic technologies. We begin by highlighting the critical role of precise control in fabricating advanced micro- and nanomaterials. The discussion then centers on fundamental platforms for microfluidic synthesis, covering various microreactors, their operational principles, and synthesis strategies, including high-throughput systems that accelerate material discovery. We introduce key classes of materials suited for microfluidic synthesis, such as polymeric and hydrogel microparticles, nanoparticles with controlled nucleation and growth, and functionalized hierarchical structures. Additionally, we examine on-chip manipulation and integration techniques that facilitate complex workflows, from synthesis and purification to quality control, enabling the on-demand production of personalized therapeutics. Looking forward, the convergence of microfluidics with artificial intelligence will create intelligent platforms capable of self-optimizing control. Finally, we address the current challenges of microfluidic-based synthesis and offer perspectives on future developments in this innovative field.

## 2. Fundamental Microfluidic Platforms for Synthesis

### 2.1. Droplet-Based Microfluidics: Monodisperse Microreactors

**Figure 1:**
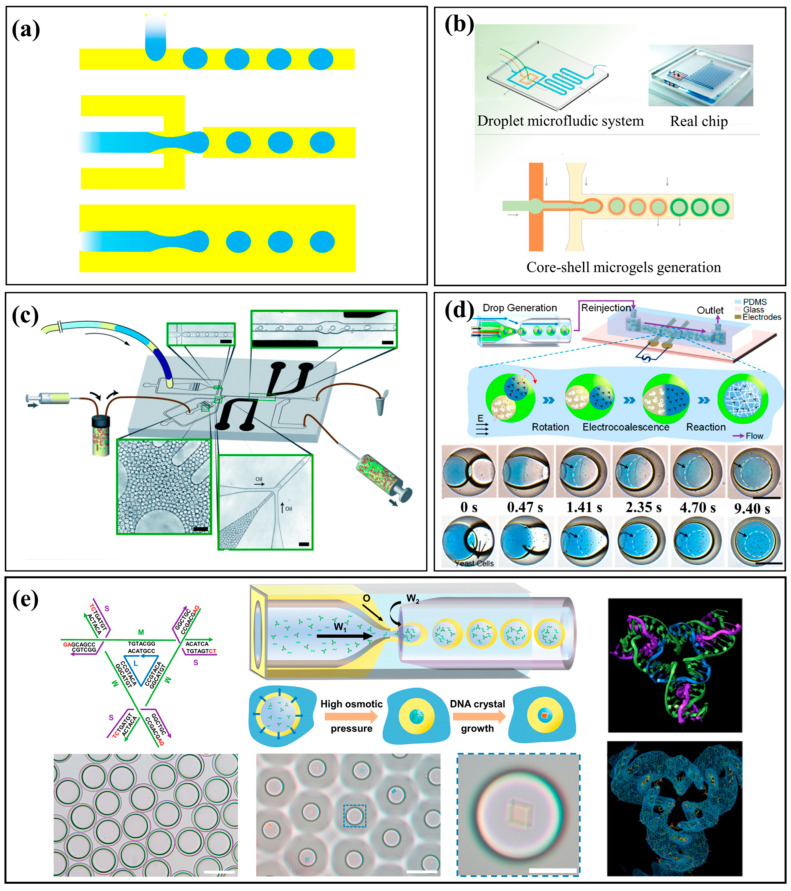
Droplet-based microfluidics. (**a**) Droplet generation using three types of microfluidic structures: cross-flow, flow-focusing, and co-flow. (**b**) One-step droplet microfluidic method to generate core–shell GelMA microgels [44]. Reprinted with permission from ref. [44]. Copyright 2019 Wiley. (**c**) A droplet-based microfluidic platform for picolitre-scale combinatorial synthesis enabled by an applied electric field [45]. Reprinted with permission from ref. [45]. Copyright 2012 Royal Society of Chemistry. (**d**) A microfluidic strategy for continuous microreactions by applying external AC electrical signals to trigger core coalescence within double-emulsion droplets [46]. Reprinted with permission from ref. [46]. Copyright 2017 American Chemical Society. (**e**) A microfluidic approach using double-emulsion droplets as nanoliter-scale reactors to precisely control the self-assembly of monodisperse 3D DNA crystals [47]. Reprinted with permission from ref. [47]. Copyright 2025 American Chemical Society.

Droplet-based microfluidics transforms a continuous stream into discrete droplets by leveraging interfacial tension between immiscible fluids [35]. This process generates picoliter-to-nanoliter-sized droplets that serve as isolated reaction vessels and can be precisely loaded, transported, and manipulated within the microfluidic system [36,37]. Common droplet generation geometries include cross-flow (e.g., T-junction), flow-focusing, and co-flow structures (Figure 1a) [39,43]. In droplet-based microfluidics, geometry strongly influences droplet size, uniformity, and flexibility. T-junctions are simple and robust but sensitive to flow fluctuations. Flow-focusing ensures high uniformity and precise size control, ideal for high-throughput or encapsulation. Co-flow, with the dispersed phase coaxial to the continuous phase, gently forms droplets with minimal shear, suitable for fragile cells or biological cargo, though uniformity is lower. Understanding these trade-offs guides selection of structures based on throughput, size control, and fluid compatibility. For example, Wang et al. developed a one-step method to generate core–shell GelMA microgels with tunable size and high biocompatibility, offering a promising platform for tissue engineering and organoid generation (Figure 1b) [44]. External fields can also be used to manipulate droplets. Theberge et al. created a platform for picoliter-scale combinatorial synthesis where an applied electric field induced electrocoalescence to fuse reagent-containing droplets, enabling efficient generation of small-molecule libraries (Figure 1c) [45]. Similarly, Hou et al. used external AC electrical signals to trigger core coalescence within double-emulsion droplets for continuous microreactions and hydrogel fabrication (Figure 1d) [46]. More recently, this technology has been applied to synthesize advanced biomolecules. Chen et al. used double-emulsion droplets as nanoliter-scale reactors to precisely control the self-assembly of monodisperse 3D DNA crystals, achieving high yield and uniform size (Figure 1e) [47]. By confining reactants within uniform droplets, this approach offers precise control over chemical reactions, improves reaction speed, and enhances product consistency while reducing reagent consumption. The integration of external electric and magnetic fields has further refined control over reaction timing, paving the way for complex, multistep syntheses of advanced functional materials.

### 2.2. Continuous-Flow Microfluidics: High-Throughput Synthesis Streams

**Figure 2:**
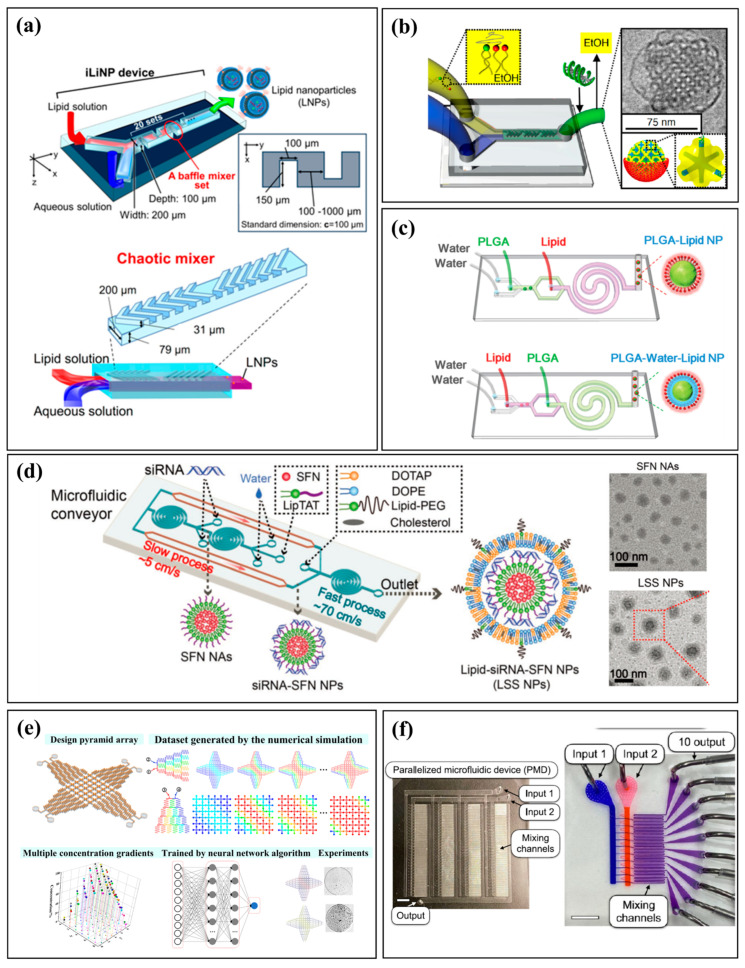
Continuous-flow microfluidics: high-throughput synthesis streams. (**a**) A baffle mixer device and the chaotic mixer device [49]. Open access and uses a Creative Commons public use license. (**b**) Microfluidics synthesis of gene silencing cubosomes [50]. Reprinted with permission from ref. [50]. Copyright 2018 American Chemical Society. (**c**) A two-stage microfluidic platform precisely controls the sequential mixing to fabricate polymer–lipid hybrid nanoparticles [48]. Reprinted with permission from ref. [48]. Copyright 2014 Wiley-VCH. (**d**) A microfluidics-enabled serial assembly platform that precisely controls sequential steps to fabricate lipid–siRNA–sorafenib nanoparticles [4]. Reprinted with permission from ref. [4]. Copyright 2023 Wiley-VCH. (**e**) Two-dimensional pyramid-array microfluidic chip capable of generating controllable multiple concentration gradients [51]. Reprinted with permission from ref. [51]. Copyright 2023 American Chemical Society. (**f**) Microfluidic high-throughput integration in the efficient synthesis of lipid nanoparticles [52]. Reprinted with permission from ref. [52]. Copyright 2021 American Chemical Society.

Continuous-flow microfluidics involve the constant pumping and mixing of fluids within a single-phase stream. The high-precision fluid manipulation capabilities of these systems enable precise control of experimental parameters—such as flow rates, concentration and temperature—to ensure consistent material size and morphology. This continuous operation allows for high-throughput production. Common microfluidic geometries include T-shaped, Y-shaped, spiral and staggered herringbone micromixers [38]. T- and Y-shaped designs are widely used due to their simple fabrication, but they rely on molecular diffusion, resulting in low mixing efficiency and broad particle size distributions. To overcome this, advanced channel structures have been developed to enhance mixing without increasing system complexity [48]. For example, spiral and staggered herringbone micromixers improve reaction homogeneity by inducing secondary flows and chaotic advection, which increase the interfacial area between fluids and facilitate rapid, uniform mixing. This enhanced mixing improves the kinetics of solvent exchange and component distribution. Kimura et al. developed a baffle mixer that demonstrated more effective fluid blending than a standard chaotic mixer, enabling the synthesis of smaller, more consistent lipid nanoparticles under identical flow conditions (Figure 2a) [49]. Similarly, Kim et al. developed a platform with structurally enhanced chaotic mixing to produce uniform cubosomes as small as 75 nm, overcoming the size limitations of traditional methods and advancing the design of effective gene delivery vectors (Figure 2b) [50]. The synthesis of functional nanoparticles thus critically relies on precisely controlling flow dynamics and reactant assembly within the microfluidic chip. Efficient synthesis of structurally complex nanoparticles has been advanced by designing systems that enhance mixing and integrate multiple processes onto a single platform. For instance, Sun et al. developed a two-stage microfluidic device that precisely controls sequential mixing to fabricate polymer-lipid hybrid nanoparticles with tunable rigidity by adjusting the injection order of solutions (Figure 2c) [48]. Chen et al. designed a serial assembly platform that functions like a mini-conveyor to control the stepwise fabrication of lipid-siRNA-sorafenib nanoparticles, enabling continuous production of multifunctional nanosystems with high encapsulation efficiency (Figure 2d) [4]. Such structurally enhanced mixing leads to more reproducible synthesis conditions and yields nanoparticles with narrower size distributions. This precision is particularly advantageous for multicomponent systems requiring sequential assembly of functional layers, such as drug cores and lipid shells.

High-throughput strategies are essential for unlocking the full potential of microfluidics in material synthesis. By integrating precise control over reaction conditions with high-throughput methods, these systems accelerate the discovery of functional materials. For example, Qi et al. designed a two-dimensional pyramid-array chip that generates multiple controllable concentration gradients for high-throughput, multiplexed reactions, as validated by crystallization experiments (Figure 2e) [51]. This design enables continuous, large-scale material synthesis and screening, which is critical for developing nanomaterials, polymers, and biomaterials. The modular nature of these systems also makes them suitable for industrial-scale production. Shepherd et al. demonstrated this scalability with a 128-channel parallelized device that achieves over 100-fold higher throughput in lipid nanoparticle production, providing a robust platform for manufacturing RNA therapeutics and vaccines (Figure 2f) [52]. Their silicon-glass microfluidic platform employs parallelized generating units, enabling precise nanoparticle formulation with significantly enhanced production capacity. By combining precise fluid control with channel parallelization, the system improves mixing efficiency and reproducibility [53]. By enabling precise control and parallel processing, high-throughput microfluidic synthesis significantly accelerates material discovery and optimization while minimizing reagent consumption.

### 2.3. Microfluidic Synthesis Enabled by External Fields

**Figure 3:**
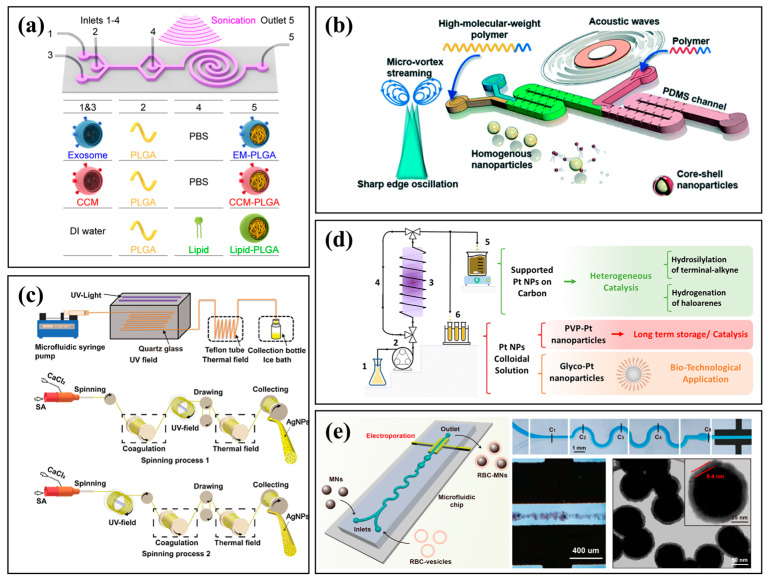
Microfluidic synthesis enabled by external fields. (**a**) Microfluidic synthesis integrated with an acoustic field [54]. Reprinted with permission from ref. [54]. Copyright 2019 American Chemical Society. (**b**) Acoustofluidic synthesis platform [55]. Reprinted with permission from ref. [55]. Copyright 2021 Royal Society of Chemistry. (**c**) Microfluidic synthesis coupled with ultraviolet/thermal fields [56]. Reprinted with permission from ref. [56]. Copyright 2025 American Chemical Society. (**d**) Photo-induced microfluidic synthesis [57]. Reprinted with permission from ref. [57]. Copyright 2024 Royal Society of Chemistry. (**e**) Microfluidic electroporation-facilitated synthesis [58]. Adapted with permission from ref. [58]. Copyright 2017 American Chemical Society.

Microfluidic synthesis enhanced by external fields provides a versatile platform for precision microscale chemical processing. By incorporating electric, magnetic, acoustic, or thermal fields, researchers can actively regulate fluid dynamics, particle manipulation, and reaction kinetics in real time. These stimuli improve mixing efficiency, enable selective transport, and allow dynamic tuning of reaction conditions, which is particularly beneficial for synthesizing complex nanomaterials and smart drug delivery systems. For example, Liu et al. used an external acoustic field to efficiently assemble membrane-coated nanoparticles, simplifying fabrication while improving immune evasion and tumor-targeting efficiency (Figure 3a) [54]. Similarly, Zhao et al. engineered an acoustofluidic platform that enables ultrafast micromixing to synthesize size-tunable, high-molecular-weight polymeric nanoparticles with core–shell structures (Figure 3b) [55]. Other fields have also been integrated: Liu et al. coupled ultraviolet and thermal fields to fabricate AgNP-loaded alginate fibers with enhanced uniformity and antibacterial performance (Figure 3c) [56]; Marelli et al. developed a photo-driven method to produce ultrasmall (~1.3 nm) platinum nanoparticles as stable, efficient catalysts (Figure 3d) [57]; and Rao et al. used electroporation to synthesize erythrocyte membrane-coated magnetic nanoparticles with improved stability and circulation (Figure 3e) [58]. The synergy between microfluidic confinement and external physical fields enhances mixing, selectivity, and product uniformity, opening new avenues for automated synthesis and scalable manufacturing.

## 3. Microfluidic Synthesis of Advanced Materials

### 3.1. Polymeric and Hydrogel Microparticles

Polymer and hydrogel microparticles, typically with diameters ranging from 1 to 1000 μm [59,60], are commonly synthesized using droplet-based microfluidic techniques that offer precise control over particle size and uniformity [30]. In these systems, monomer or polymer precursor solutions are compartmentalized into discrete droplets that serve as microreactors. Polymerization or crosslinking is initiated within these droplets by external stimuli, such as ultraviolet (UV) light [61], thermal triggers [62,63], or chemical initiators [64], enabling the efficient and reproducible fabrication of particles with tailored properties.

**Figure 4:**
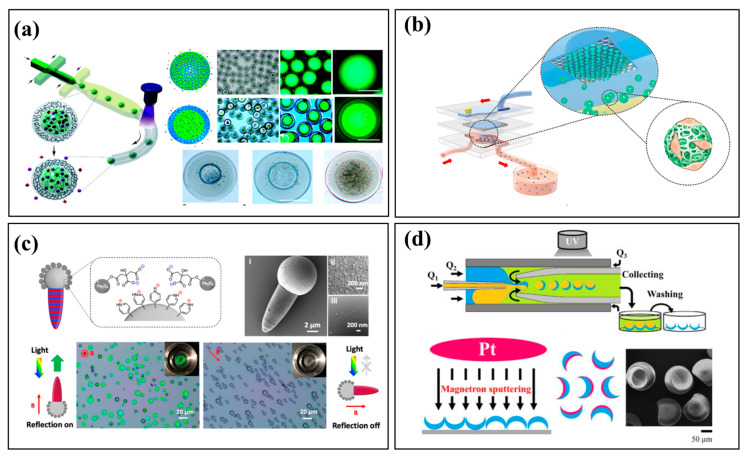
Polymeric and hydrogel microparticles. (**a**) Core–shell microcapsules fabricated [67]. Reprinted with permission from ref. [67]. Copyright 2020 Royal Society of Chemistry. (**b**) Microfluidic production of droplets and hydrogel microspheres [68]. Reprinted with permission from ref. [68]. Copyright 2023 American Chemical Society. (**c**) Multifunctional photonic Janus particles [69]. Reprinted with permission from ref. [69]. Copyright 2022 American Chemical Society. (**d**) Droplet-based synthesis of bowl-shaped microparticles from the Janus microdropletsin a capillary microfluidic device [70]. Reprinted from [70], with the permission of AIP Publishing.

Core–shell microparticles, which consist of an inner core encapsulated within a functional outer shell [65], can be fabricated with high precision using microfluidics to control the flow of core and shell phases [20,66]. This approach offers superior control over particle size, core-to-shell ratio, and material composition compared to conventional methods. For example, Dinh et al. fabricated reservoir microcapsules that overcome the typical trade-off between hydrogel size and drug-loading capacity (Figure 4a) [67], while Wu et al. developed a high-throughput system to generate uniform hydrogel microspheres for biomedical applications (Figure 4b) [68]. Microfluidics also enables the synthesis of complex morphologies, such as the multifunctional photonic Janus particles reported by He et al. (Figure 4c) [69] and the bowl-shaped micromotors with precisely controlled Pt-coated surfaces developed by Wang et al. (Figure 4d) [70]. Furthermore, external fields can be integrated for advanced particle manipulation. Jin et al. used a focused surface acoustic wave (FSAW) platform to create multilayered core–shell microcapsules by manipulating particles with acoustic radiation forces [71]. Owing to their customizable properties and biocompatibility, polymeric and hydrogel microparticles are widely used in biomedicine. They can be engineered as drug delivery vehicles for controlled and sustained release [72], as multifunctional platforms for cell delivery and tissue scaffolding [73], or as encoded particles for multiplexed bioassays, improving the efficiency and scalability of diagnostic platforms. Polymeric and hydrogel microparticles are widely used in biomedical fields due to their customizable properties and excellent biocompatibility.

### 3.2. Nanoparticles: Mastering Nucleation and Growth

**Figure 5:**
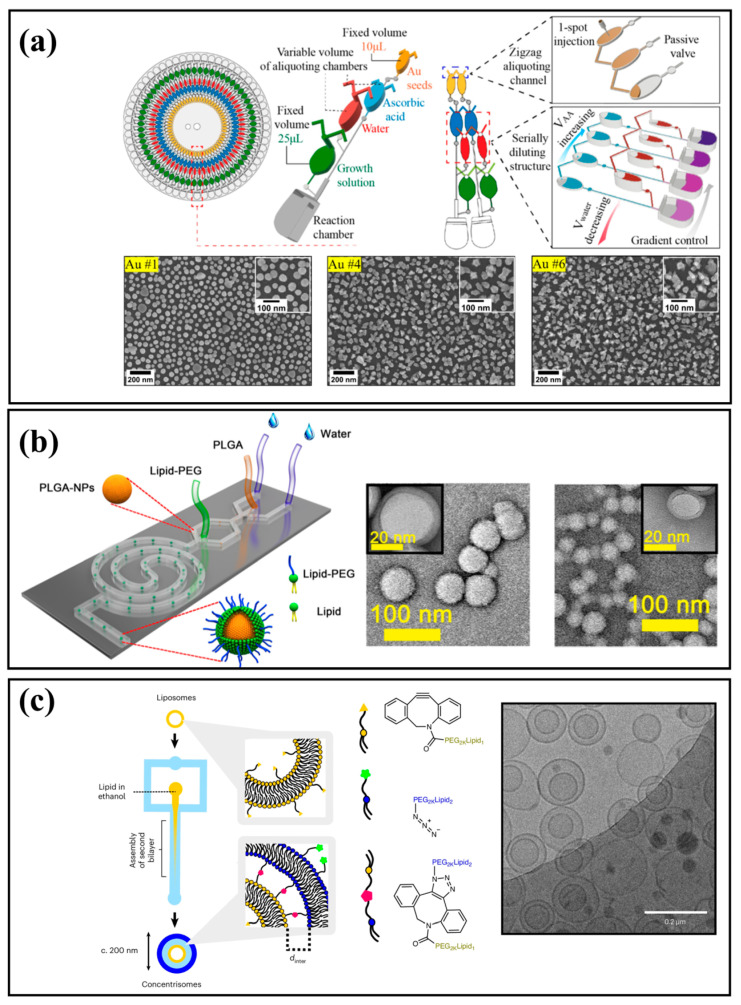
Nanoparticle synthesis and applications. (**a**) Synthesis of gold nanoparticles with tunable morphologies [74]. Reprinted from Chemical Engineering Journal, Vol 452/Part1, Hiep Van Nguyen et al., Serially diluting centrifugal microfluidics for high-throughput gold nanoparticle synthesis using an automated and portable workstation, Pages No.139044, Copyright (2022), with permission from Elsevier. (**b**) Synthesis of monodisperse lipid–PLGA hybrid nanoparticles [78]. Reprinted from [78], with the permission of AIP Publishing. (**c**) Nanoscale liposome-in-liposome particles [79]. Open access article distributed under the terms of the Creative Commons CC BY license.

Achieving uniform nanoparticle size and morphology relies on precise control over nucleation and growth dynamics. Continuous-flow microfluidic systems are the predominant method for this, as they enable rapid and consistent mixing for controlled particle formation, while droplet-based techniques offer isolated environments for reproducible parallel synthesis. By modulating temperature gradients and reagent concentrations, microfluidic platforms can temporally and spatially separate particle formation from subsequent growth, leading to highly uniform nanoparticle populations with narrow size distributions. This precise control is effective for synthesizing various inorganic nanoparticles. For example, Nguyen et al. developed an automated centrifugal platform for the high-throughput synthesis of gold nanoparticles with tunable morphologies via 60 parallel reactions on a single chip (Figure 5a) [74]. Okatenko et al. demonstrated that voltage-driven reactions in microfluidic setups enable the controlled synthesis of liquid Ga–metal nanoparticles by regulating oxide reduction [75]. Similarly, Hu et al. reported a one-step microfluidic synthesis of biofunctionalized CuInS2/ZnS quantum dots with tunable near-infrared emission for targeted bioimaging [19]. For polymeric and lipid nanoparticles, the primary advantage of microfluidics is its precise control over solvent exchange and self-assembly, which enables narrow size distributions, tunable dimensions, and high-efficiency drug loading [38,76,77]. For example, Feng et al. developed a high-throughput platform to synthesize monodisperse lipid–PLGA hybrid nanoparticles with tunable sizes (Figure 5b) [78]. Pilkington et al. engineered “concentrisomes,” nanoscale liposome-in-liposome particles with two distinct compartments for multi-stage release (Figure 5c) [79]. Firmino et al. used a 3D-twisted microfluidic device for high-mass production of monodisperse nanoliposomes [80], while Zheng et al. showed that the topology of lipid nanoparticles critically enhances endosomal escape and RNA delivery [81]. As shown in Table 1, microfluidic synthesis is a precise and efficient strategy for generating uniform nanoparticles with finely controlled size, shape, and composition. This enhances their value in diagnostics and drug delivery [3,26,82]. PLGA nanoparticles have broad biomedical applications, especially in targeted cancer therapy and bioimaging. For example, AS1411 aptamer-modified PLGA nanoparticles that carry polyphyllin II induce apoptosis in hepatocellular carcinoma cells [83], while dual-drug PLGA carriers that co-deliver curcumin and niclosamide improve therapeutic outcomes in breast cancer [84]. Curcumin-loaded PLGA nanoparticles have also enabled luminescent imaging [85], demonstrating their potential for therapy and diagnosis. Lipid nanoparticles can encapsulate hydrophilic and lipophilic agents [86] and enable targeted delivery [87]. Surface engineering improves stability [88], uptake, and controlled release. Metal nanoparticles function as nanocarriers for drug delivery, enhancing circulation, stability and efficacy [89,90]. Quantum dots offer unique advantages for cancer imaging and drug delivery [91,92,93], with tunable optical properties, surface functionalization, and dual diagnostic and therapeutic potential [94]. Microfluidic synthesis reproducibly fabricates these diverse nanoparticles with uniform, optimized characteristics, thereby strengthening their biomedical performance.

As summarized in Table 1, microfluidic synthesis offers finely controlled and tunable size distributions, reduced dispersity, and scalability across different material systems. For example, PLGA nanoparticles show uniformity with high encapsulation efficiency; lipid nanoparticles achieve sub-100 nm sizes with scalable production; metallic nanoparticles benefit from consistent morphology; and quantum dots provide tunable optical and electronic properties. These broad advantages highlight the versatility and practical benefits of microfluidic platforms. However, challenges in scalability and process integration remain. Further advances in device design are needed to unlock the full potential of microfluidics for large-scale nanoparticle production.

Mixing time is a critical parameter in nanoparticle synthesis because it affects the balance of nucleation, growth, and aggregation. Bulk and microfluidic studies consistently demonstrate that shorter mixing times produce smaller, more uniform nanoparticles, while slower mixing promotes heterogeneous growth [39]. Importantly, the ratio of mixing time to the characteristic aggregation or nucleation timescales determines whether controlled self-assembly can be achieved [34]. Since different fabrication strategies produce different mixing times, this parameter is a valuable comparative metric:

Hydrodynamic Flow Focusing (HFF): Enables ultrafast mixing that promotes highly uniform and reproducible nanoparticles with precise control over particle size and consistency. The mixing time (*τ_mix_*) in HFF can be approximated as [106], $\tau_{mix}∼\frac{w_{f}^{2}}{4D}\approx \frac{w^{2}}{9D}\left(\frac{1}{(1+1/R{)}^{2}}\right)$, where *D* is the solvent diffusivity, *w_f_* is the width of the focused stream, *w* is the width of the channel, and *R* is the ratio of flow rate of the polymeric stream to the total flow rate of water.

Passive Micromixers (lamination, herringbone, etc.): The mixing performance depends on the channel geometry and the flow conditions. For example, staggered herringbone microstructures (SHM) can induce chaotic advection, achieving millisecond-scale mixing. This enables the rapid self-assembly of siRNA-loaded liposomes with well-controlled size and narrow distribution [107].

Droplet-Based Microfluidics: Internal recirculation within droplets accelerates solute homogenization, yielding mixing times of a few milliseconds [108]. This approach provides fast, uniform mixing and offers compartmentalization to reduce cross-contamination and enable precise size control.

Bulk or Batch Mixing: Typically involves mixing times on the order of seconds, leading to poor size control and broad distributions compared to microfluidics.

To facilitate comparison across different fabrication strategies, Table 2 summarizes various microfluidic mixing mechanisms, their characteristic timescales, and their principal advantages in nanoparticle formation.

### 3.3. Functionalized Materials and Hierarchical Structures

Functionalized materials are engineered with specific chemical groups that enable tailored interactions with their environment, enhancing properties like selectivity, responsiveness, and biocompatibility [109]. Synthesizing these materials requires precise control over spatial organization and chemical heterogeneity, which is challenging with traditional batch methods that suffer from poor uniformity and limited tunability. Microfluidic synthesis overcomes these limitations by offering highly controlled reaction environments. For instance, Jaradat et al. used a microfluidic approach to fabricate PEGylated paclitaxel-loaded liposomes with over 90% encapsulation efficiency, demonstrating that flow rate ratios and PEG-lipid content critically influence particle properties [110]. Microfluidic platforms enable the generation of functionalized materials with well-defined characteristics and support the fabrication of hierarchical architectures through techniques like templating and layer-by-layer assembly. By controlling flow dynamics and reaction kinetics, microfluidics allows for the reproducible production of complex materials, including core–shell nanoparticles, Janus particles, and porous scaffolds.

Microfluidic technology offers a highly controlled method for synthesizing metal–organic frameworks (MOFs), overcoming limitations of bulk synthesis like poor size uniformity and low reproducibility. The microscale environment enables rapid mixing, efficient heat and mass transfer, and precise control over reaction conditions, allowing for the continuous production of MOF crystals with consistent morphology. This provides a scalable platform for developing high-quality MOFs for catalysis and drug delivery. For example, Huang et al. synthesized hierarchical conductive MOF (c-MOF) films with hollow interiors and nanoporous shells that facilitate directional mass transport and enhance interfacial mass transfer [111]. In a similar study, they enhanced mass transport in heterogeneous catalysis by encapsulating Pd nanocubes within deformable MOF nanosheets that facilitate dye adsorption under fluidic shear forces [112]. As versatile nanomaterials with tunable structures, MOFs have diverse applications in drug delivery, imaging, and other therapeutic approaches [113].

The supramolecular assembly of block copolymers into defined nanostructures like micelles and vesicles depends on precisely controlled environmental conditions. For instance, templating with bicontinuous block copolymer assemblies can form porous materials with ordered cubic architectures (1–500 nm pore size), achieving exceptional structural uniformity [114]. In drug delivery, surfactant-block copolymer hybrids self-assemble into core–shell micelles whose size, membrane thickness, and payload are dictated by parameters such as amphiphile ratios and solvent conditions. For complex miktoarm star copolymers, an evaporation-induced microenvironment modulated by deep learning allows the targeted formation of diverse morphologies by tuning topological asymmetry and curvature [115]. These studies demonstrate that by finely controlling variables such as composition, solvent conditions, and flow, researchers can reliably direct block copolymer self-assembly, offering a powerful platform for engineering nanoscale materials.

Microfluidic synthesis offers a precise and scalable route for fabricating functionalized materials and hierarchical structures [116], overcoming key limitations of conventional batch methods like poor uniformity and low reproducibility. By enabling fine control over flow dynamics, concentration gradients, and reaction kinetics, microfluidics facilitates the production of complex architectures with defined morphology and surface chemistry. This level of control is essential for tailoring material performance in applications such as drug delivery and catalysis. The ability to integrate templating, droplet manipulation, and layer-by-layer assembly within confined microenvironments further enhances the precision and versatility of the resulting materials, positioning microfluidics as a powerful platform for next-generation materials design.

Although treated as distinct categories, microparticles, nanoparticles, functionalized materials, and hierarchical structures are closely interrelated within the framework of microfluidic synthesis. Polymer and hydrogel microparticles, typically at the micrometer scale, provide structural versatility and biocompatibility, making them suitable for applications such as drug delivery and tissue engineering. In contrast, nanoparticles are prized for their tunable physicochemical properties, high surface-area-to-volume ratios, and capacity for intracellular delivery. Functionalized materials build upon these concepts by introducing chemical or structural modifications that confer selective responsiveness or enhanced interfacial functionality, while hierarchical structures integrate multiple length scales and functionalities into unified architectures. These material classes form a continuum of structural complexity that can be systematically engineered through microfluidic platforms.

## 4. On-Chip Manipulation and Integration

### 4.1. Post-Synthesis Sorting and Purification

Post-synthesis separation is a critical step in materials fabrication, as reactions often yield complex mixtures of particles with diverse sizes, shapes, and compositions. Efficient purification is essential for enhancing material performance and enabling scalable applications. While traditional methods like centrifugation are effective in some contexts, they are often limited by low efficiency, poor resolution, and difficulty in automation. Microfluidic separation technologies offer significant advantages, including minimal sample consumption, high resolution, and continuous operation [117,118,119]. Integrating synthesis with on-chip separation creates unified platforms for the one-step acquisition of target materials, establishing a fully integrated “synthesis-to-analysis” workflow that enhances material screening and development. Microfluidic separation techniques can be classified as passive or active.

Passive methods rely on channel geometry and fluid dynamics to separate particles based on intrinsic properties like size and deformability. These techniques are simple, stable, and well-suited for large-scale screening. Common passive methods include:

Inertial microfluidics, which uses inertial lift forces in curved or serpentine channels to separate particles by size at high throughput [120,121,122,123].

Viscoelastic microfluidics, which utilizes elastic forces in non-Newtonian fluids to achieve superior separation of smaller particles, such as nanoparticles and viruses [124,125,126,127,128,129,130,131].

Deterministic lateral displacement (DLD), which uses an array of micropillars to achieve high-precision separation with predictable cutoff sizes [132].

Microfluidic filtration, which employs micro-porous structures or slits for continuous particle enrichment and purification [133].

Active methods use external fields (e.g., electric, magnetic, acoustic, or optical) to exert controlled forces on particles, enabling precise manipulation with high tunability and resolution. Common active methods include:

Electric field-based techniques, such as dielectrophoresis, which are widely used for precise sorting [134,135,136,137,138,139,140,141,142].

Magnetic separation, which selectively isolates magnetic or magnetically tagged particles [143].

Acoustic methods, which use ultrasound to manipulate particles in a non-contact and gentle manner, ideal for sensitive materials [144,145].

Optical trapping, which uses light to achieve highly precise localization and sorting [146,147].

Recent advancements in microfluidics have significantly enhanced the manipulation and separation of nanoscale particles, particularly those smaller than 100 nm. Zhang et al. introduced acoustoelectronic nanotweezers, which utilize electronic and acoustic fields to dynamically control sub-100 nm nanoparticles with minimal disturbance [144]. Zeng et al. developed a label-free, biocompatible on-chip magnetic separation system, achieving high recovery and purity of nanoscale particles [143]. Hettiarachchi et al. optimized viscoelastic microfluidics to improve submicron particle separation by tuning flow rates and polymer concentrations, achieving a separation resolution of up to 100 nm [129]. Additionally, Asghari et al. presented sheathless oscillatory viscoelastic microfluidics, enabling efficient focusing and separation of nanoscale species, including particles below 100 nm [148]. These techniques offer precise, scalable, and biocompatible approaches for nanoscale particle manipulation, advancing applications in nanotechnology and biomedicine.

By rationally selecting and integrating these passive and active strategies, microfluidic separation has become an indispensable tool for automated material development and quality control.

### 4.2. Towards Integrated “Synthesis-to-Analysis” Platforms

Integrated “synthesis-to-analysis” platforms represent a key direction in microfluidic synthesis. These systems integrate synthesis, sample processing, and analysis on a single chip, enabling automated workflows with minimal reagent consumption, which improves efficiency and reduces human error. For example, Lu et al. developed a platform combining automated photocatalytic synthesis and high-throughput screening that allows the exploration of up to 10,000 reaction conditions per day [149]. Li et al. created a similar platform for screening ligand interactions during the synthesis of cesium lead bromide nanocrystals to rapidly optimize their photoluminescence properties [150]. Li et al. present Microfluidic Print-to-Synthesis (MPS), a high-throughput platform for automated peptide microarray synthesis with low reagent use. The system supports multiplexing and scalable design. A peptide library was screened on live cells. MPS arrays are spatially addressable, enabling direct peptide identification, making it a powerful tool for rapid peptide screening and optimization [151]. More broadly, the core advantage of integrated “synthesis-to-analysis” platforms lies not only in their physical integration but also in the reconfiguration of experimental logic. The core advantage of these platforms is the reconfiguration of experimental logic from a linear workflow to a closed-loop system capable of autonomous optimization [152]. These systems generate products, perform in situ characterization, and feed the results back into a control algorithm for iterative optimization. Future development will focus on building automated, closed-loop platforms that integrate synthesis, characterization, and functional screening with real-time biosensing modules to enable intelligent monitoring and adaptive control, thereby accelerating new material discovery.

## 5. Challenges and Future Outlook

### 5.1. Overcoming Current Hurdles

The primary challenge for industrial-scale microfluidic synthesis is the throughput bottleneck. Unlike traditional chemical synthesis, which scales up by increasing reactor size, simply enlarging microfluidic channels compromises the precise hydrodynamic control that defines the technology. This “numbering-up” approach can reduce mixing efficiency, lead to uneven mass transfer, and alter fluid shear forces, ultimately sacrificing product quality and reaction consistency. A more promising strategy is “scaling out” by parallelizing a large number of identical microreactor units. This approach preserves the advantages of microscale reactions while increasing production capacity. However, scaling out introduces its own challenges, including ensuring uniform flow distribution, preventing clogging, and managing complex control systems, all of which increase fabrication cost and complexity. Therefore, advancing scalable microfluidic synthesis requires the development of low-cost, standardized, and highly integrated parallel platforms. Researchers are exploring strategies using microfluidic technologies to address throughput limitations and scale up the synthesis of functional materials for industrial applications. One notable development is the platform introduced by Shepherd et al., which integrates up to 256 parallel lipid nanoparticle-generating units on a single silicon-glass microfluidic chip [53]. Its modular architecture allows for flexible scaling and demonstrates compatibility with pharmaceutical manufacturing standards. In the field of inorganic nanomaterials, Geng et al. developed a nanoliter droplet-based microfluidic reactor that can continuously synthesize perovskite nanocrystals on a large scale with highly tunable optical properties by controlling the ratios of the precursors, the temperature, and the residence time [153]. Coliaie et al. presented an advanced, continuous-flow microfluidic device that enables the rapid, parallel screening of crystal polymorphs, morphologies, and crystallization kinetics under precisely controlled supersaturation conditions. This device greatly accelerates the development of crystalline materials [154]. These innovations collectively demonstrate the transformative potential of microfluidic platforms in overcoming scalability challenges in material synthesis.

Due to their small channel dimensions, microfluidic systems are prone to clogging and fouling, which challenges their long-term operational stability. The accumulation of particles, precipitates, or bubbles can lead to blockages and flow instability. Overcoming the challenges of particle deposition, surface fouling, and bubble accumulation is essential to ensuring stable microfluidic operation. Mitigation strategies include surface modifications, such as hydrophilic coatings, to reduce particle adsorption; optimized channel geometries to improve particle and bubble removal; and droplet-based microfluidics to encapsulate reactants and minimize contact with channel walls. Eder et al. demonstrated that covalently immobilizing lubricants on PDMS effectively reduces particle adhesion [155], and Hao highlighted liquid-like interfaces that sustain drag reduction and minimize fouling [156]. Regarding bubbles, Ren et al. demonstrated that ultrasonic cavitation can efficiently detach adherent bubbles and prevent blockage [157]. Additionally, Li et al. developed ultrathin polymer brush coatings that provide durable antifouling protection [158]. These advances are important steps toward overcoming the persistent difficulties of clogging and instability in microfluidic systems.

Looking ahead, microfluidic systems must evolve from complex laboratory setups into robust, standardized, and user-friendly “plug-and-play” devices. This requires highly integrated designs that combine fluid control, reaction monitoring, and feedback regulation on a single chip, reducing reliance on external equipment. Standardized and modular designs are essential for improving reproducibility and ensuring stable, long-term operation. Furthermore, intelligent software for automated process control will be critical for reducing operational complexity and driving the transition of microfluidics from the laboratory to industrial and clinical applications.

### 5.2. The Future of Microfluidic Synthesis

The future of microfluidic synthesis lies in fully automated and intelligent systems. By integrating real-time sensors with machine learning algorithms, “self-optimizing” platforms can dynamically adjust synthesis parameters to maximize product quality and efficiency, accelerating the development of new materials by eliminating time-consuming trial-and-error approaches. Several emerging directions are expected to play an increasingly important role in the future development of microfluidics. For instance, 3D-printed microfluidic platforms enable rapid and customizable device fabrication with reduced cost and design constraints [159,160,161]. By tailoring chip geometries and flow conditions, researchers have achieved precise control over lipid nanoparticle size [162], developed customizable devices for drug screening and tissue engineering [163], and enabled seedless, surfactant-free synthesis of tunable gold nanostars using acoustically enhanced mixing [164]. Flexible and wearable microfluidics open opportunities for continuous health monitoring and personalized diagnostics, thereby extending applications beyond the laboratory [165,166,167]. In the context of sustainable and green synthesis, microfluidic continuous-flow systems have been applied in diverse applications, ranging from magnetic field-assisted biocatalytic reactors for furfurylamine production [168], to microreactor-based strategies that improve the efficiency and selectivity of rare-earth extraction and separation [169], and to continuous-flow devices that enable the environmentally benign assembly of bio-derived nanostructures such as cardanol–cholesterol complexes [170]. Future platforms will also evolve from single-step reactions to integrated, multi-step processes. Complex chips will incorporate modules for synthesis, purification, functionalization, and quality control, enabling a complete workflow on a single device. For example, a chip could continuously synthesize drug delivery nanoparticles, purify them, functionalize their surface, and test their performance in an automated sequence, enhancing efficiency and consistency for industrial-scale production. This advancement in miniaturization and intelligence will enable point-of-use manufacturing, particularly for personalized medicine. This model allows for the on-site preparation of patient-specific drugs or short-lived radioactive tracers, reducing logistical challenges and improving response times, especially in remote areas. Recent advances in integrating machine learning with microfluidic and automated synthesis platforms have enabled precise control over advanced nanomaterials [171,172,173,174,175,176]. Machine learning-assisted microfluidic liposome synthesis, for instance, has achieved broad-spectrum size regulation [177], with ensemble models accurately predicting both particle size and polydispersity index across varying solvents and flow conditions. In curcumin-loaded liposome production, supervised models such as support vector machines and neural networks efficiently identified optimal lipid concentrations, flow ratios, and drug loading levels, reducing experimental workload while ensuring formulation stability [178]. Extending beyond lipid systems, machine learning-guided nanoparticle synthesis demonstrates the power of hybrid strategies: two-step frameworks combining Bayesian optimization with deep neural networks allow targeted control of optical properties in silver nanoparticles [179], and closed-loop platforms integrating robotics with machine learning dynamically adjust reaction parameters to rapidly converge on optimal product features [180]. Despite these advances, challenges remain in dataset quality and the translation of laboratory-scale microfluidic processes to industrial production. Finally, by leveraging high-throughput screening and AI-driven data analysis, automated microfluidic systems will rapidly explore vast reaction parameter spaces to accelerate the discovery of novel functional materials, driving innovation in fields from catalysis to biomedicine.

## 6. Conclusions

Microfluidic technologies are a powerful strategy for synthesizing advanced functional materials. These technologies enable precise control over microscale reaction environments. Lab-on-a-chip platforms facilitate the fabrication of polymeric, hydrogel, lipid-based, inorganic, and hierarchical nano- and microparticles with exceptional uniformity, tunability, and functionalization. This level of control over particle size, morphology, and composition improves drug encapsulation, controlled release, targeting, and delivery efficiency. These improvements are critical for applications in drug delivery, diagnostics, catalysis, and nanomaterial engineering. Microfluidics is particularly beneficial for reactions involving expensive reagents and multifunctional materials. Innovations in channel design, chip stacking, and integrated purification are paving the way for scalable production from the laboratory to industrial settings.

This review provides a comprehensive overview of microfluidic platforms for materials synthesis. Droplet-based microfluidics uses monodisperse microreactors to confine reactants and minimize reagent use. In contrast, continuous-flow systems support high-throughput synthesis with accelerated kinetics. At the microscale, microfluidics improves the uniformity and morphology of polymeric and hydrogel microparticles for drug delivery and assay applications. At the nanoscale, rapid mixing yields inorganic, polymeric, and lipid-based nanoparticles with narrow size distributions. These platforms also enable functionalization, hierarchical structuring, and efficient screening of reaction conditions. Comparative evaluations guide optimal fabrication strategies, and integrated on-chip analysis supports multistep “synthesis-to-analysis” workflows.

Looking ahead, the future of microfluidic synthesis lies in integration, automation, and AI-driven optimization. Self-optimizing systems with real-time feedback can fine-tune synthesis conditions, accelerate discovery, and improve reproducibility. Meanwhile, sustainable practices that minimize reagent waste and adopt environmentally friendly protocols are becoming increasingly important. The development of robust, standardized, and user-friendly “plug-and-play” devices will promote wider adoption in manufacturing and biomedicine. Overall, microfluidic synthesis is a versatile, precise, and scalable platform with transformative potential in nanotechnology, materials science, personalized medicine, and industrial applications.

## Figures and Tables

**Table 1:** A comparison of different nanoparticle types.

Type	Microfluidic Methods and Controlled Parameters	Advantages	Reference
PLGA (Polymeric) NPs	A hydrodynamic flow-focusing microfluidic method was employed to fabricate PLGA nanoparticles, with key parameters including flow rate ratio, total flow rate, and polymer/surfactant concentrations, enabling high encapsulation efficiency and sustained, pH-dependent drug release.	Z-average size of 128 ± 8 nm (PDI *<* 0.2), ζ-potential of −14.8 ± 5.3 mV and high encapsulation efficiency (98.6 ± 5.8%).	Bai et al. [95]
	An ultrasonic microreactor was used to synthesize PLGA nanoparticles by emulsion-solvent evaporation, with key parameters—ultrasonic power, PLGA concentration, and flow rate ratio—optimized to control particle size and uniformity.	PDI of 0.1–0.2, 115–150 nm	Udepurkar et al. [96]
	A microfluidic iLiNP device was used to precisely tune PLGA nanoparticle sizes (40–114 nm) by adjusting flow rates, enabling size-controlled sub-200 nm drug-loaded nanoparticles without changing polymer precursors.	PLGA NPs: 44–101 nm; PEG-PLGA NPs: 29–76 nm; blend NPs: 40–114 nm	Bao et al. [97]
Lipid NPs (LNPs)	Chaotic microfibrous channels enable continuous lipid nanoparticle production via multiple phase-splitting, with smaller fiber diameters and higher continuous-phase flow rates yielding smaller, more uniform particles.	89.7 ± 35.1 and 190.4 ± 66.4 nm	Ahn et al. [98]
	A glass-based piling-up microfluidic device system was developed, enabling controlled RNA-loaded lipid nanoparticle production at high flow rates (20–50 mL/min) with particle sizes of 20–60 nm for scalable mass manufacturing.	20 and 60 nm at a flow rate of 20–50 mL/min	Maeki et al. [99]
	3D-printed ring micromixers with controllable flow rate and ring asymmetry enable high-throughput production of size-controlled, monodisperse lipid nanoparticles with efficient mRNA encapsulation.	Diameters less than 90 nm, low polydispersity (<0.2), and high mRNA encapsulation efficiency (>91%)	Hong et al. [100]
	A stereolithography-fabricated 3D-printed microfluidic device using omnidirectional sheath flow and a staggered herringbone mixer enables high-throughput (60 mL min^−1^) production of mRNA-loaded lipid nanoparticles.	Diameter less than 90 nm, with low polydispersity (2–8%) and high mRNA encapsulation efficiency (>90%).	Lin et al. [101]
Metallic NPs	A high-throughput centrifugal microfluidic platform integrated with a portable automated workstation enables 60 parallel gold nanoparticle syntheses.	120.5 nm, 117.3 nm, and 114.1 nm in diameter	Nguyen et al. [74]
	A seed-mediated in situ synthesis method was implemented in microfluidic reactors, where flow rate and channel geometry were identified as key parameters influencing gold NPs growth, morphology, and surface coverage.	Nanostar, 60 nm~100 nm	Vinnacombe-Willson et al. [102]
Quantum Dots	A microfluidic Pickering emulsion method was developed to synthesize uniform magnetic/fluorescent microspheres with tunable optical barcoding, using droplet size control, silica nanoparticle stabilization, and quantum dot encapsulation for multiplex tumor marker detection.	High-throughput ultrasensitive detection, the detection limits of 0.027 ng/ mL for CEA, 1.48 KU/L for CA125 and 1.09 KU/L for CA199	Li et al. [103]
	A magnetic-field-coupled microfluidic method was used to synthesize Co-doped ZnSe quantum dots, where varying magnetic fields (0–100 mT) controlled doping level, particle size, and band gap, thereby tuning their magnetic and optical properties.	Co-doped ZnCoSe quantum dots (~4–6 nm)	Zhao et al. [104]
	A microfluidic dripping technique was employed to fabricate acrylamide polymer microspheres doped with AgInS2/ZnS quantum dots, controlling droplet formation via flow rates and channel design parameters.	Mean value of the decay time for quantum dots in solutions is 91 and 3.5 ns	Kurassova et al. [105]

**Table 2:** A comparison of mixing mechanisms, timescales, and key features in microfluidic nanoparticle formation.

Mixing Mechanism/Geometry	Typical Mixing Time	Key Features/Advantages
Hydrodynamic Flow Focusing	$\tau_{mix}∼\frac{w_{f}^{2}}{4D}\approx \frac{w^{2}}{9D}\left(\frac{1}{(1+1/R{)}^{2}}\right)$	Good size control; narrow size distribution; smaller particles; high encapsulation efficiency for drug delivery; reproducible.
Passive Micromixers (e.g., lamination, staggered herringbone)	Depending on channel design and flow rates. SHM can achieve chaotic mixing within milliseconds.	Good mixing without external fields; relatively simple devices; lower energy/ lower complexity.
Droplet-Based Microfluidics	Rapid solute homogenization via internal circulation (some reported a few milliseconds).	Excellent compartmentalization; reduced cross-contamination; control over individual reaction “chambers”; improved mixing via internal flows.
Active Micromixers (acoustic, electrical, etc.)	Some active mixers achieve mixing times faster than passive counterparts (i.e., lower ms), though exact reported values depend on device.	Mixing can be tuned; high efficiency even at higher flow rates; can reduce required channel length; may reduce residence time.

## Data Availability

No new data were created or analyzed in this study.
